# Non-invasive laparoscopic detection of small tumors of the digestive tract using inductive sensors of proximity

**DOI:** 10.1038/s41598-022-04822-x

**Published:** 2022-01-14

**Authors:** Adrian Calborean, Sergiu Macavei, Mihaela Mocan, Catalin Ciuce, Adriana Bintintan, Adrian Cordos, Cosmin Pestean, Romeo Chira, Liviu Zarbo, Lucian Barbu-Tudoran, George Dindelegan, Felix Nickel, Bogdan Mocan, Valeriu Surlin, Vasile Bintintan

**Affiliations:** 1 National Institute for Research and Development of Isotopic and Molecular Technologies, Donath Street, No 67-103, Cluj-Napoca, Romania; 2 Iuliu Hatieganu University of Medicine and Pharmacy Cluj-Napoca, V. Babeş Street No. 8, 400012 Cluj-Napoca, Romania; 3 University of Agricultural Sciences and Veterinary Medicine of Cluj-Napoca, Calea Manastur Street No. 3-5, Cluj-Napoca, Romania; 4 Clinic for General, Visceral and Transplantation Surgery, University of Heidelberg, INF 110, Heidelberg, Germany; 5 Technical University Cluj-Napoca, Memorandumului, Street No.28, Cluj-Napoca, Romania; 6 University of Medicine and Pharmacy Craiova, Petru Rares Street No. 2, 700115 Craiova, Romania

**Keywords:** Biomedical engineering, Oncology, Surgical oncology

## Abstract

The precise location of gastric and colorectal tumors is of paramount importance for the oncological surgeon as it dictates the limits of resection and the extent of lymphadenectomy. However, this task proves sometimes to be very challenging, especially in the laparoscopic setting when the tumors are small, have a soft texture, and do not invade the serosa. In this view, our research team has developed a new instrument adapted to minimally-invasive surgery, and manipulated solely by the operating surgeon which has the potential to locate precisely tumors of the digestive tract. It consists of an inductive proximity sensor and an electronic block encapsulated into an autoclavable stainless-steel cage that works in tandem with an endoscopic hemostatic clip whose structure was modified to increase detectability. By scanning the serosal side of the colon or stomach, the instrument is capable to accurately pinpoint the location of the clip placed previously during diagnostic endoscopy on the normal bowel mucosa, adjacent to the tumor. In the current in-vivo experiments performed on large animals, the modified clips were transported without difficulties to the point of interest and attached to the mucosa of the bowel. Using a laparoscopic approach, the detection rate of this system reached 65% when the sensor scanned the bowel at a speed of 0.3 cm/s, and applying slight pressure on the serosa. This value increased to 95% when the sensor was guided directly on the point of clip attachment. The detection rate dropped sharply when the scanning speed exceeded 1 cm/s and when the sensor-clip distance exceeded the cut-off value of 3 mm. In conclusion, the proposed detection system demonstrated its potential to offer a swift and convenient solution for the digestive laparoscopic surgeons, however its detection range still needs to be improved to render it useful for the clinical setting.

## Introduction

Due to larger availability of endoscopic diagnosis^1,2^ and implementation of screening programs for gastric and colorectal cancer^3,4^, the incidence of small, early gastric, and colonic tumors that are referred for surgical treatment has increased significantly. Although these tumors are the ones most suitable for a minimally-invasive surgical approach (MIS)^5^, precise intraoperative identification of their position is difficult in laparoscopy since they are not visible from the serosal side while the haptic feedback offered by the laparoscopic instruments is significantly less reliable than palpation is for open surgery^1,2,6^.

Currently, location of the tumor is approximated by integrating the length measured endoscopically from the tumor to the anal verge or dental arcade with findings from imaging radiology (computed tomography-CT or magnetic resonance imaging -MRI)^3,4^. However, the endoscopic measurement of distances is fairly estimated because the colon is elongated and distended by insufflation during endoscopy, while small tumors are difficult to be visualized on CT or MRI.

Endoscopic tattooing^7,8^ and intraoperative endoscopy^9^ are the techniques most commonly used to aid in intraoperative location of these tumors. Both have their own disadvantages. The first requires compliance from the endoscopist, and has certain limitations related to the quality of the dye, depth of injection, or widespread staining of the surrounding tissues if injected intraperitoneally^10–12^. The latter is largely dependent on the availability of an endoscopic device in the operative theatre and is mainly performed in modern hybrid operating rooms^13^. Moreover it implies distension of the bowel with residual gas which impedes adequate visualisation during the subsequent stages of laparoscopic resection. Intraoperative ultrasound may be helpful in identifying the tumor, but it has limited sensibility, requires appropriate and expensive high-end devices, an experienced radiologist present into the operative theatre and, in MIS is furthermore restricted by the fixed position of the trocars^14^.

Use of indocianin green as fluorescent marker attached to the jaws on standard endoscopic clips was also proposed in a clinical trial^15^. The method allowed precise identification of the tumor in 6 of a total of 8 patients and looks quite promising. However it has the disadvantage of poor penetration of tissue, in the range of 2 mm, by the near infrared fluorescence especially with vertical illumination. It also requires an atypical near-infrared fluorescence imaging system to detect and display the image of the marked clips.

Another interesting solution is described by Wada et al.^16^ which attached a light emmiting diode to a coiled antenna creating a 9 × 2 × 2 mm device that could pass through the forceps aperture of a gastrointestinal endoscope and was deployed to the mucosa attached to a standard hemostatic clip. The system allowed detection of the clip in all 3 patients participating to the trial, however, there are still issues with transportation of the LED-antena units and their long-term attachment on the mucosa of the colon.

Although the last reports are quite promising, the proposed methods are still in an experimental phase and require high-end devices. Given the actuality of the problem in our clinical practice and the lack of a convenient solution, we have tried to find an alternative approach to intraoperative tumor location during MIS, which avoids the disadvantages of the above-mentioned options. We also aimed to reduce the number of specialists and expensive devices required for detection, within the aim of increasing the utility of the technique in hospitals which have less advanced equipment and staff. In this respect, we have designed and constructed a sensing instrument compatible with laparoscopic surgery, capable to detect metallic tags placed endoscopically on the mucosa of the digestive tract (colon, stomach) in the vicinity of the tumor within days or weeks before surgery, ideally at the time of the diagnostic endoscopy. By detecting these metallic tags, the surgeon is aware of the exact position of the tumor and thus can plan the resection accordingly.

The system was presented already in our recent articles^17,18^, in which we have intensively tested its functionality in ex-vivo dry and wet lab experiments, on synthetic materials, biologic tissues and human surgical specimens. In this work, the primary aim was to evaluate the feasibility of the proposed detection system in “in-vivo” experiments, in conditions similar with the ones encountered in the operative theatres. The second aim of the study was to evaluate if the attachment to the deployment mechanism, and attachment of the clips on the mucosa, are influenced by the process of metal deposition.

## Materials and methods

### Tumor detection principle

**Figure 1:**
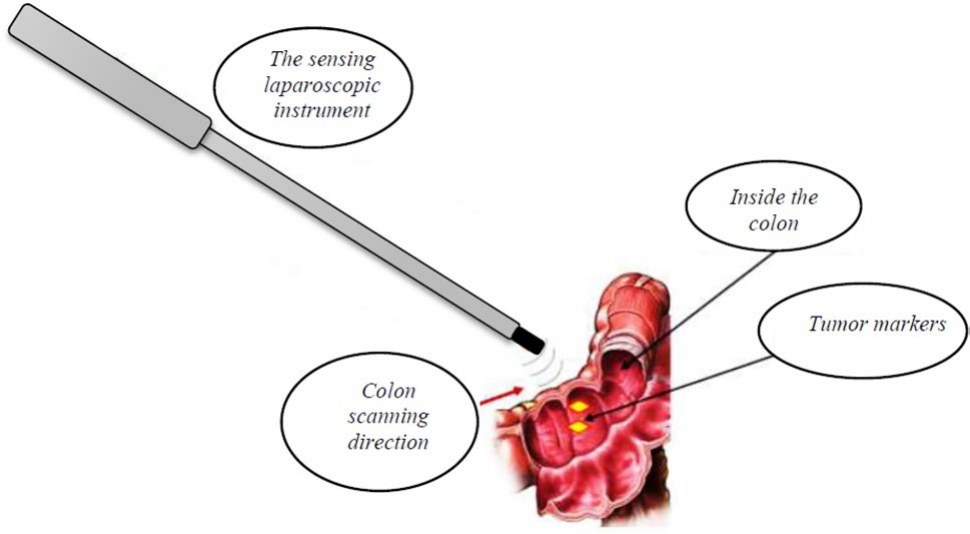
Principle of tumor detection by the complex formed from the sensing laparoscopic instrument and the endoscopically attached metallic tags—tumor markers.

The principles of tumor localization can be briefly described as follows: a modified endoscopic hemostatic clip serving as metallic tag is placed endoscopically on the mucosal side of the gastric or colonic wall in the vicinity of the tumor at the time of diagnostic endoscopy or later, ideally between two weeks and two days before surgery. Multiple clips can be applied around the periphery of the tumor if desired. During the laparoscopic operation, the sensing instrument scans the serosa of the bowel in the segment where the tumor is supposed to be located based on measurements recorded in the endoscopic report and on information provided by sectional imaging. When the instrument detects one clip an acoustic signal is produced and the tumor is precisely located (see Fig. 1).

In a particular manner, the inductive proximity sensors detect the presence of hemostatic metallic coated clips entering into their oscillating field, providing a target detection. The system contains an oscillator which creates a high frequency electromagnetic field that is radiated from the coil in the sensor front area.

When this field contacts the conducting coated clips, a small current is induced within the hemostatic clips, which further generate their own electromagnetic field that interferes with the field originating from the coil. This causes a modification in the amplitude of the oscillations of the signals from the coil system. The output voltage should be calibrated to this modification.

### Construction of the detecting laparoscopic instrument

The detection instrument has been used inductive proximity sensors that have the capability to identify presence of metallic tags. In an extensive previous research work^17^, five types of instruments incorporating different inductive proximity sensors^19–23^ were constructed and extensively tested and optimized, with the aim to develop, verify and adopt the most appropriate clinical protocols for tumor detection. The process of development and construction, from watertight encapsulation of the inductive proximity sensor into stainless steel rods sheaths to fabrication of the electronic operation block module and elaboration of the swift connection between these two components is presented elsewhere^17,18^. The instrument was designed using a modular platform that allows widely-available sterilisation by autoclavation without damaging the electronic module.

In summary, the inductive proximity sensor is hermetically mounted on the distal end of a stainless-steel rod which resembles a laparoscopic instrument by having an outer diameter of 12 mm and a length of 45 cm. The 12 mm diameter was chosen to accommodate larger, 10 mm diameter sensors, which have more potent detection capabilities but it also has the disadvantage that it requires the larger, 12.5 mm laparoscopic trocars for introduction into the abdominal cavity. The junction between the sensor and the stainless-steel rod is hermetically sealed with a resin to withstand multiple sterilisations. The proximal end of the detection instrument has a length of 18.5 cm, is composed of a stainless-steel case which serves as handle for the instrument and was designed to comply with the ergonomics and manipulation properties required by the surgical team. It incorporates the electronic functional block and is hermetically closed with a metallic lid to ensure proper sterility. The electronic functional block was dimensionally scaled-down to fit into the stainless-steel case while the instrument was constructed using a modular platform that allows disconnection of the electronic block and sterilization of the sensor-case unit by autoclavation (45 min at 121 °C and 1.2 bar pressure) followed by swift plugging of the electronic block just before surgery in a sterile-controlled environment.

**Figure 2:**
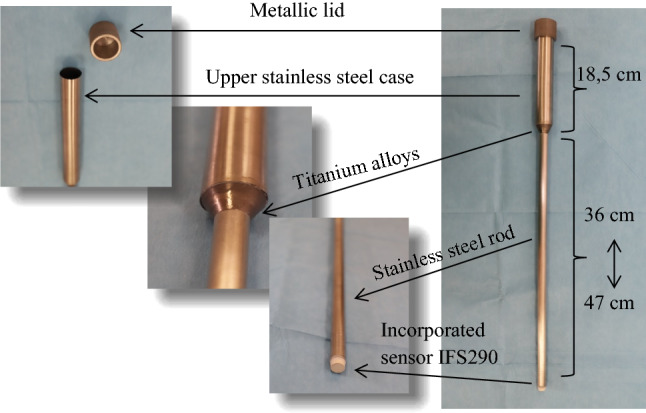
The modular laparoscopic detection instrument composed from a proximal handle that hosts the electronic block covered by a lid to preserve an sterile environment on the surface (corner left), the titanium alloy at the junction between the case and the rod (middle left), the inductive sensor present at the distal tip of the instrument, encapsulated in a watertight junction (middle right). Overview of the whole instrument (right).

The updated version of our instrument is shown in Fig. 2 right. The Teflon connection head (incorporating gold electrical connectors) was replaced with a titanium alloy connection head in an attempt to avoid any risk of microbial retention (Fig. 2—middle left). The electronic block can be now easily removed by opening the metallic lid (Fig. 2—left), and the equipment (Fig. 2—right) can be sent for sterilisation following the standard autoclaving protocol.

Based on our previous research work^17^ performed in gaseous (air) and liquid (0.9% NaCl, 5% Glucose solutions, etc.) environments, and continued on ex-vivo surgical specimens (animals and human)^18^, we have concluded that the 12 mm laparoscopic detecting system that incorporates a 10 mm diameter proximity sensor (IFS290 and E59M12C110C02-D1), offers the longest detection range and will be used in the “in-vivo” experiments. Both sensors, use a built-in microprocessor to provide smallest detection and errors that can be modified from the microprocessor software to intelligently detect and maximize sensor performance depending on the environment used. They are capable to perform these actions with accurate monitoring of very small changes in field saturation when the target moves in the detection field, then compares these changes to specific user-programmed values.

### Construction of custom-made metallic clips

The conventional endoscopic hemostatic clips (Olympus HX-610-9090L and Olympus HX-610-135L) were chosen as metallic tags that should be detected by the sensing instrument, due to their large availability and possibility to be deployed anywhere along the digestive tract using standard endoscopic instruments and techniques. In order to increase the detectability of these clips, they were further engineered by surface depositions of nanometric layers of cooper (Cu), zinc (Zn) and gold (Au) in various layer thicknesses from 10 nm, 20 nm and 30 nm, and then increasing every 100 nm up to a maximum of 1 micron. The plastic side of the clip was protected by a custom-made shield.

Deposition of thin layers of Cu, Zn and Au was done through Physical Vapor Deposition (PVD) technique. In this process, the material passes from a condensed phase to a vapor phase, and then back to a condensed thin film form. The targets used had a purity of 99.998%. Cu and Zn were chosen in an attempt to improve the detection range while Au was coated at the end of each deposition proccess (deposition thickness values of 50 nm) to ensure biocompatibility of the modified clip. Although their thicknesses have been intensively tested up to 1 micron, the detection randament decreases exponentially after 300 nm thickness with about 0.2 mm per 100 nm added thickness, regardless of the environment in which the first measurements were performed.

Previous experiments performed by our research team on a multitude of clip combinations coated with various concentrations of Cu, Zn and Au, showed that the longest detection range was associated with the followig combinations: (1) Cu 200 nm + Au 10 nm; (2) Cu 300 nm + 48 Zn + Au 10 nm and (3) Cu 500 nm + 20 nm Au. Therefore, in the following in-vivo experiments, only these modified clip variants were used.

### Deployment of custom-made metallic clips during “in-vivo” experiments

**Figure 3:**
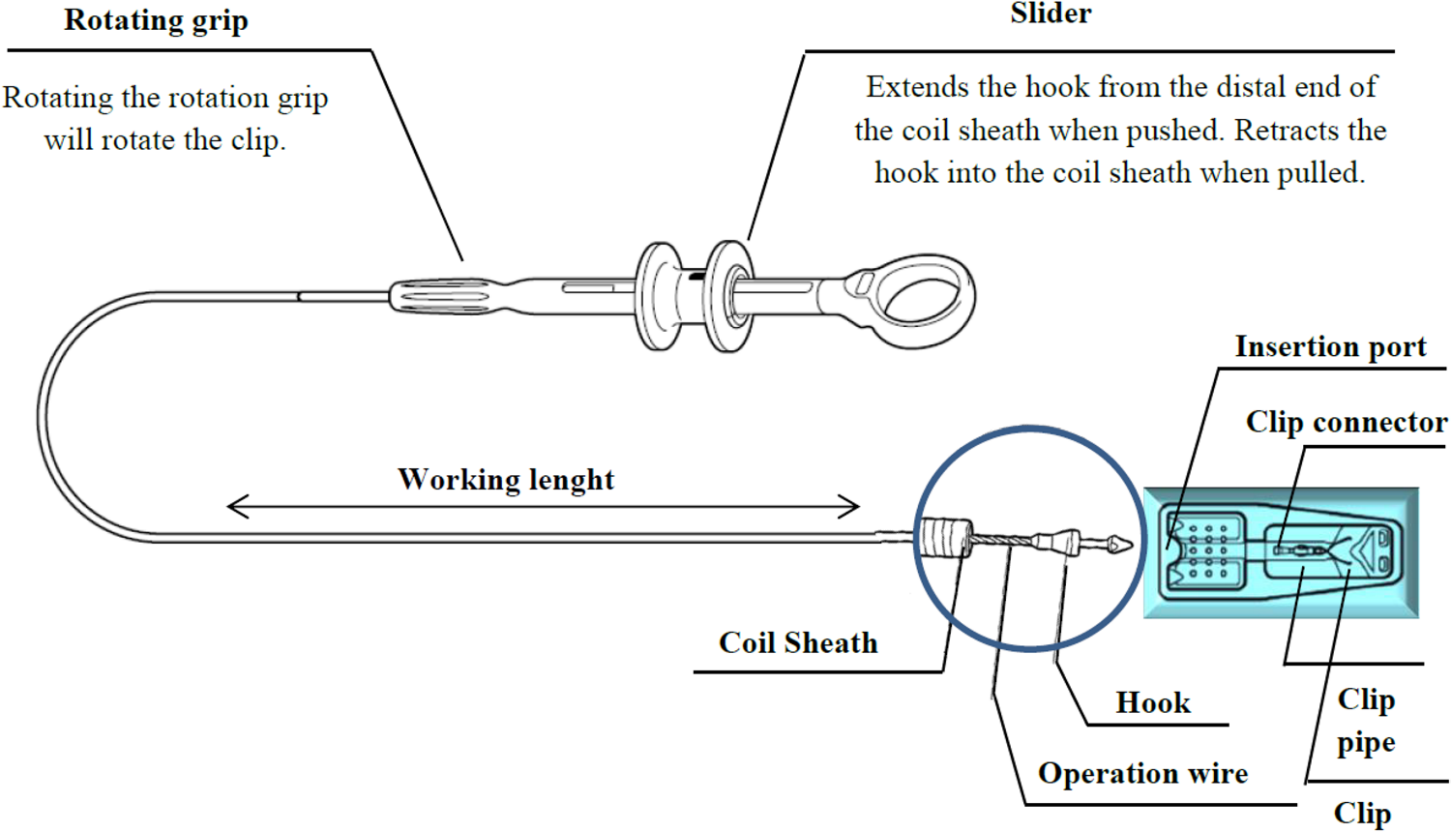
System for endoscopic deployment of modified clip at the point of interest into the gastrointestinal tract.

The Karl Storz™ flexible endoscope with a 2.8 mm diameter working channel was used for the transport and deployment of the clip to the point of interest. The modified clip was placed into the standard cover of the usual Olympus HX-610-9090L or Olympus HX-610-135L hemostatic clips. The attachment tip of the clip, which is made of plastic, was not modified by the vapour deposition process and was supposed to fit easily into the hook of the operation wire. The clip was thus attached to the wire and carefully withdrawn into the working channel of the endoscope. Observations were made if the modified clip fitted inside the working channel and was able to be transported to the site of interest on the mucosa. Once the location of clip deployment was chosen, the slider was moved distally and the clip was deployed using a procedure similar with deployment of a standard hemostatic clip. The process is schematically drawn in Fig. 3. The success rate of clip deployment and attachment on the mucosa was also recorded.

### “In-vivo” preparation and testing set-up of the detection system

**Figure 4:**
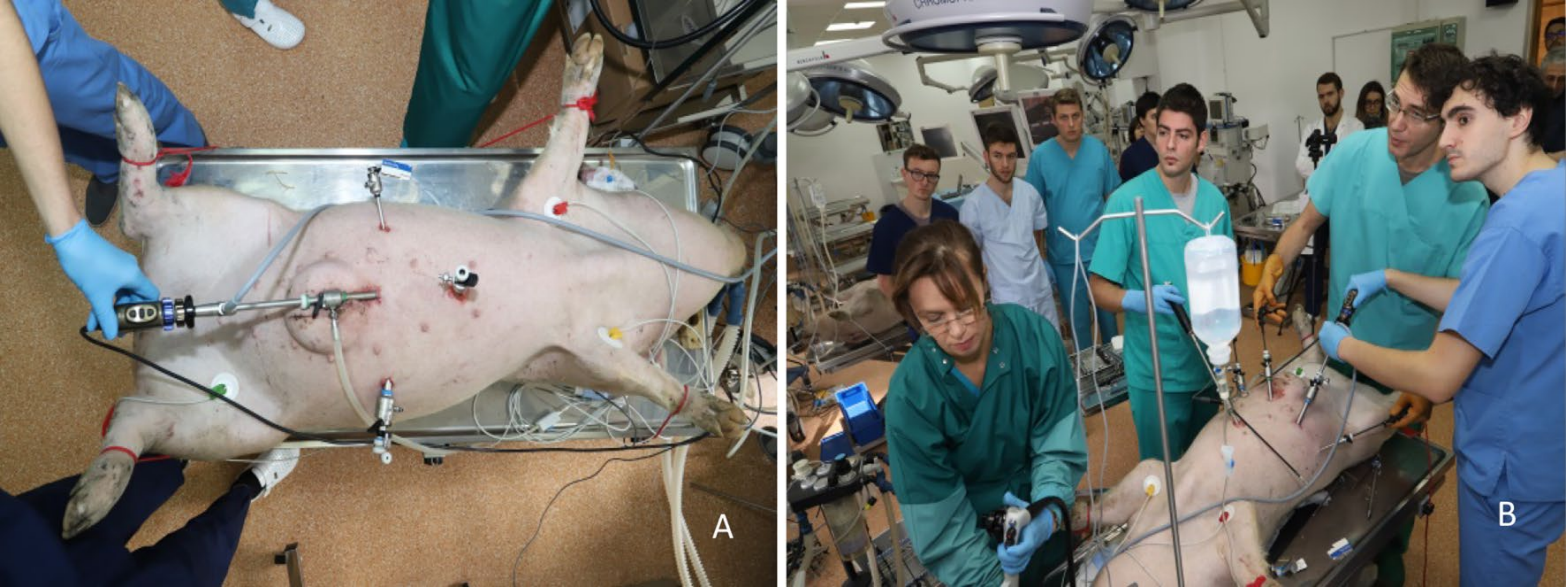
(**A**) Laparoscopic surgical access to the peritoneal cavity; (**B**) trans-oral insertion of the clip and deployment into the duodenum.

The functionality and sensitivity of the detection system system was evaluated in “in-vivo” experiments performed on large animals. For these experiments were used two pigs with weights of 60 and 65 kg, respectively, operated laparoscopically in deep anaesthesia (Fig. 4). After premedication with azaperone (1–2 mg/kg, intramuscular [IM]) and midazolam (0.5–0.7 mg/kg, IM), narcosis was induced with midazolam (1–1.5 mg/kg, intravenous [IV]), ketamine (10 mg/kg, IV), and atropine sulfate (0.05 mg/kg IV). Animals were intubated and anaesthesia was maintained using an isoflurane enriched O_2_/air mixture and N_2_O. Fentanyl (500 µg/h, IV) was used for analgesia and pancuronium (0.25 mg/kg/h) for muscle relaxation. The common carotid artery and internal jugular vein were surgically catheterized and connected to membranous pressure transducers for continuous measurement of mean arterial pressure and central venous pressure, respectively. Ringer lactate solution was infused continuously during the operation at a rate of 20 Ml/kg/h. Heart rate and rhythm were monitored by a surface electrocardiogram.

Experiments were carried out respecting the European conventions and ARRIVE guidelines for animal welfare and were authorized by the decision of the Ethical Committee of the University of Medicine Cluj-Napoca No 156/02.04.2018. We also attest to informed consent for publication of identifying information/images in online open-access publication.

In order to prepare the colon mechanically to create adequate conditions for intraoperative colonoscopy, animals were fed with 2 L of diluted polyethylene glycol solution (Fortrans) in the last 24 h before surgery and 4 h preoperatively with trans rectal instillation of one packet of Clismalax solution.

Surgical access to the peritoneal cavity was obtained through a laparoscopic approach (Karl Storz, Tutlingen, Germany) using three trocars, one 10 mm trocar for the optics, one 5 mm trocar for an atraumatic bowel grasper and a 12 mm trocar for the 12 mm detection instrument. Concomitantly, the endoscopy team loaded the modified clip into the applicator of the flexible Karl Storz™ endoscope using the instrumentation and technique described in the previous chapter (Fig. 4A). The endoscope and the attached clip were advanced trans-anal into the upper rectum and the sigmoid where the clip was deployed on the mucosa of the colon. Both standard^24^ and modified hemostatic clips were used for the experiment, the former ones acting as the control group. The clips were spaced at a distance of at least 5 cm from each other, the location of the clips being determined by the endoscopist. In one experiment the clip was placed through a trans-oral route into the duodenum using the same approach (Fig. 4B).

**Figure 5:**
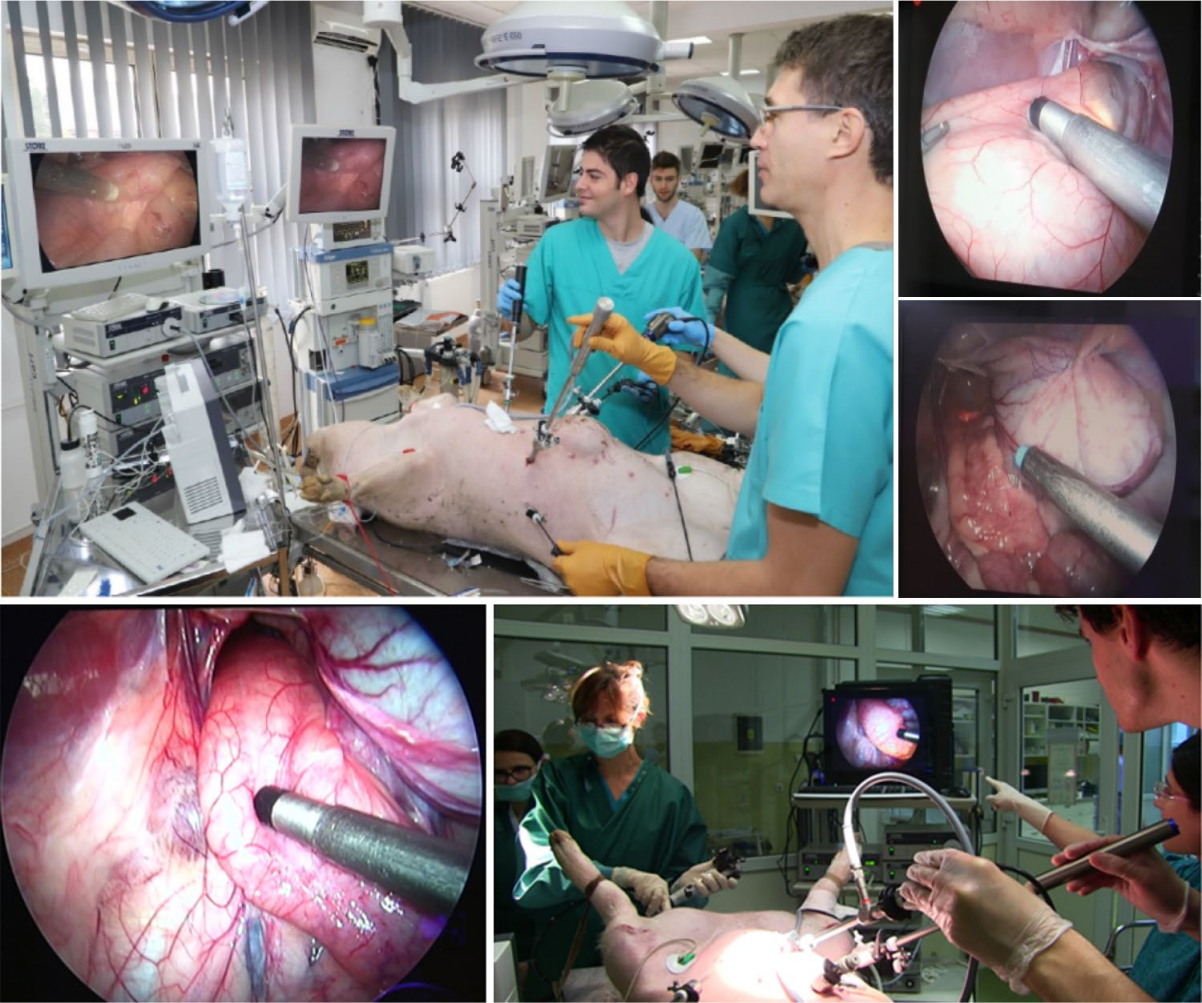
Combined laparoscopic and endoscopic approach to identify the position of the clip with zoom on instruments scanning clip position.

The main investigator from the surgical team was blind to the exact location of the clip, he was only informed that the clip was either into the rectum or into the sigmoid, mimicking the information provided to the surgeon by the preoperative endoscopic measurements from the anal verge to the tumor. After each deployment of a clip, he attempted to discover its exact location by scanning the serosal side of the bowel (Fig. 5). Scanning was performed with the laparoscopic detector applying slight pressure on the bowel wall, initially with a speed of 1 cm/s followed by following attempts at a speed of 0.3 cm/s. In total, 10 detection attempts were performed for each clip at a speed of 0.3 cm/s speeds. In every case, at the end of those mandatory 10 detection attempts, a concomitant endoscopic/laparoscopic exploration of the bowel segment was performed to confirm that the detection signal recorded was given by the specific metallic clip searched for (Fig. 5). Furthermore, after detection, the location of the clip was marked on the serosa and the detector was again directed on that point for 10 times to record the reproducibility of the detection signal.

## Results

### Attachment and deployment of the clips

Clips were attached to the mucosa of intraperitoneal bowel segments which had a maximum wall thickness of 2.5 mm. The stomach, which has a wall thickness exceeding 3 mm, was thus excluded and so were the colon from the ileocolic junction to the sigmoid which, in the swine, is spiral and has reduced peritoneal lining. Endoscopic access to the duodenum proved to be quite difficult due to the lenght and shape of the stomach and was abandoned after the first succesful attempt. Finally, the sigmoid and upper rectum were the segments which provided easy endoscopic access and had a wall thickness in the range of 2 mm considered adequate for the scope of the research.

A total of 8 modified and 2 standard titanium clips were tested in two animals. In the first animal, 1 modified clip was introduced into the duodenum while 1 standard clip and 2 other modified clips were inserted into the rectum and sigmoid colon respectively.

In the second animal were introduced 1 standard and 4 modified clips into the rectum and the sigmoid colon, up to a distance of 30 cm from the anal verge. The minimum distance between the clips was at least 5 cm.

All modified clips coated with Cu 300 nm + Au 10 nm could be retracted into the working channel of the endoscope. They were also transported to the point of interest, released, deployed and attached to the mucosa of the intestine without significant problems. The endoscopic team did not experience differences regarding docking or deployment of the clip between the standard and the modified clips. It was thus demonstrated that covering the metallic part of the clips with nanometric layers of Cu and Au did not alter their spring mechanism or their functionality.

### Detection of the clips by the laparoscopic sensing instrument

The two unmodified titanium clips, one placed on the mucosa of the duodenum and the other on the mucosa of the rectum were not detected in any of the 10 scans for each clip. The detection signal was not obtained even when the endoscopist pinpointed the surgeon the exact position of the clip and the detector applied strong pressure on the serosa exactly above it. In conclusion, the detection rate of the standard titanium hemostatic clip was 0%.

The modified clips did trigger a detection signal. The signal was present when the detector was above the clip and applied slight pressure on the serosa. If the detector was not in contact with the serosa, there was no detection signal. The overall detection rate of the modified clips, defined as number of passages over the clip that have produced a detection signal while the detector was moving along the serosa divided to the total number of attempts, was 65%. Localization of the clip by the surgeon into the indicated area was confirmed to be correct by the endoscopist in all cases. In light of these data, the sensibility and specificity of the detection system for the upper rectum and sigmoid with a wall thickness of 2–2.5 mm were 65% and 100% respectively.

The speed of movement of the detector along the serosa of the bowel played also an important part since scanning with a speed of 1 cm/s was too fast for detection of the modified clips and needed to be reduced to 0.3 cm/s in all cases. Therefore, scanning a segment of the upper rectum and sigmoid 20 cm in length and 4 cm in width with a wall thickness of 2.5 mm had a 65% chance of precisely identifying the position of the clip within a maximum period of 132 s.

Once the exact position of the clip was detected by the surgical team after those 10 detection attempts, placing the sensor exactly on the clip and applying slight pressure produced a detection signal in 95% of the cases. This experiment confirmed thus the reliability of the detection system in the “in-vivo” setting.

When the colon was insufflated with air to confirm endoscopically that the searched clip was found, the detection signal became weaker or even disappeared if the clip was on the opposite wall of the rectum/sigmoid. The signal regained in intensity after exsufflation, highlighting that the presence of endolumenal air prevented the detector to press efficiently against the clip through the bowel wall and thus had an impact on the detection range.

## Discussion

Precise intraoperative localization of endolumenal digestive tumors is the prerequisite for a successful operation. Nowadays, we experience a large enthusiasm to increase the rate of laparoscopic colorectal and gastric procedures while screening programs diagnose an increasing number of patients in the early stages of the disease. Therefore the proportion of patients with small tumors referred for surgery increases, and the surgeons might face a real challenge localizing the lesion especially when a minimally-invasive approach is used.

The modern surgeon should ideally have a multidisciplinary training background^25^ and be provided with all necessary devices in a high-tech hybrid operating room^13^. However, the reality is that many surgeons still work in suboptimally equipped hospitals, have limited competences and often limited support from the endoscopist colleagues and, in these real-life circumstances, they still need to find the precise position of a small colorectal tumor to perform an adequate operation.

In this regard, we have designed and constructed a detection instrument adapted to minimally-invasive surgery. Its tubular shape with a diameter of 12 mm allows introduction through laparoscopic trocars and its length is similar with that of laparoscopic instruments. The device is constructed in a modular fashion to allow sterilisation of the stainless-steel case without destruction of the electronic block, the latter being swiftly attached in the OR in a sterile environment. The instrument is easy to activate and use while the entire system it quite inexpensive, does not require presence of specialized personnel or expensive devices into the OR and is completely surgeon dependant.

The detection instrument works in tandem with a metallic tag whom it detects. The principle of detection relays on the capacity of the inductive proximity sensors to detect variations into electromagnetic fields. The sensor contains an oscillator which creates a high frequency electromagnetic field. When a metallic tag is within this field, a small current is induced within it which further generates its own electromagnetic field that interferes with the field originating from the sensor. This causes a modification in the amplitude of the oscillations generatingan an output voltage can be calibrated and detected by the sensor. The sensors differ in their detection sensitivity. The ones chosen for our detection system, namely IFS290 and E59M12C110C02-D1, use a built-in microprocessor to provide the widest range of detection, even for the smallest signals. Moreover, the microprocessor software can be manipulated to intelligently detect and maximize sensor performance by developing detection capabilities depending on the environment used. One such unique feature offers the possibility to detect metal objects at a certain distance or band, while ignoring the targets which are located closer or further from the scanned area. The sensors are able to perform these actions with accurate monitoring of very small changes in field saturation when the target moves in the detection field, then compares these changes to specific user-programmed values.

The standard endoscopic hemostatic clip was chosen as the ideal metallic tag because it can be deployed anywhere in the long digestive tract of the humans using the standard endoscopic instrumentation. Data from our previous research^14,15^ showed that the standard titanium clip has weak detection properties for a sensor with an outer diameter of 10 mm, which is the maximum diameter that can be considered for a laparoscopic instrument.

The clips were modified by nanoscale metal depositions of Cu and Zn using PVD (Physical Vapor Deposition) technology which complies with the modern requirements of purity for surgical and medical implants. An outer layer of gold (Au) was ultimatelly added to ensure biocompatibility of the coated clip. The PVD coating process is also environmentally friendly, greatly reducing the amount of toxic substances produced compared to other types of conventional coating involving liquid precursors or complex chemical reactions. In addition, the resulting metallic coatings have superior hardness, durability and wear resistance. The most frequently clip used in the present experiment was the Cu 300 nm + Au 10 nm. Previous work^17^ showed that increasing the thickness of Cu deposition layers is associated with increased detection range up to a certain point followed by a paradoxal behaviour and a reduction of the detection range. By testing Cu layer thicknesses up to 1 micron, we have demonstrated that the detection range decreases exponentially after 300 nm thickness with about 0.2 mm per 100 nm added thickness, regardless of the environment in which the first measurements were performed.

The most important achievement of this research is that the laparoscopic detection system has proven its efficacy into living tissue real-life situation. In our previous works^14,15^, we have demonstrated that the system is viable and detects the metallic tag in the dry and wet laboratory experiments, both on ex-vivo animal tissue and on human surgical specimens. However, the questions remained if the modified clip can actually be hooked to the transport system and deployed by attachent to the colonic/duodenal mucosa on the point of interest using the standard endoscopic instrumentation. The present study demonstrated that the proposed system is working. Adding nanometer layers of Cu on the metallic part of the clip and covering it with other nanometes layers of Au for biocompatibility did not change the spring-lock mechanism of the plastic part of the clip and did not increase the size of the clip to the extent of not entering the 2.8 mm diameter working channel of the flexible endoscope. The modified clip was attached to the endoscope and deployed on the mucosa in 100% of the attempts.

The second important question referred to the ability of clip detection in an “in-vivo” setting. In this respect, our study has a clear answer: modification of the hemostatic clip by deposition of nanometric layers of Cu and Zn rendered it visible to the detector. None of the total 20 attempts to detect the standard titanium hemostatic clip in the in-vivo setting was successful. This inability to detect repeated even when the endoscopic team directed the surgical team to place the detector on the exact spot where the clip is located. The modified clip however was detected in 65% of the cases when the serosa was scanned with a speed of 0.3 cm/s and slight pressure was applied on the serosa by the detector. This figure increased to 95% of the cases when the surgeon was guided on the exact spot where the clip was attached. These results prove the reliability of detection and suggests that this system has the potential to be useful in the clinical practice. However, the detection range is still far from satisfactory. Slight pressure on the serosa is still necessary for detection. In this regard, insufflation of the colon with CO_2_ reduced the detection signal either by increasing the distance from the detector to the clip, if the clip was attached on the oposite wall, or by interfering with the possibility to press on the clip against a more rigid structure like the retroperitoneum. The actual thickness of the duodenal, rectal or sigmoidian wall is roughly 2 mm. Therefore, we can state that the “in-vivo” real life situation detection range for the proposed system is actually around the value of 2 mm. If this cut-off value is exceeded, when fat pads or thickened intestinal wall is interposed between the clip and the detector for instance, the detection rate is expected to drop significantly.

One last observation from the present experiments is that detection is discriminative; the clips are selectively identified only when the sensor is placed above them. That allows detection of individual clips and adds precision to the detection process. This property is inversely proportional with the detection range. Since the detection range is still low, around 2 mm, the discriminative capacity of detection is high. Is it expected to decrease as the detection range improve.

In conclusion, this study demonstrates that a modified endoscopic hemostatic clip can serve as a viable marker for tumor localization not only in an “ex-vivo” laboratory setting, as was demonstrated in our previous publications, but also in “in-vivo” real-life conditions. By changing its chemical structure, the clip became visible to the inductive proximity sensor and could be localized by the laparoscopic surgeon from the serosal side of the bowel during a minimally-invasive exploration, a process impossible when a standard endoscopic clip was used. However, the current detection range though viable biologic tissue of 2–2.5 mm is far from satisfactory for the clinical practice and that currently represents the major drawback of the proposed method.

An important achievement of this work though was to establish a proof of concept which will be used in the future in our quest to develop a more efficient detection system. Our aim is to increase the detection range to at least 10 mm, value considered necessary to cover the thickness of the bowel and of the adjacent fatty tissue. One solution that we are evaluating in this respect is to attach a miniaturized radiofrequency identifier tag^26^ to the modified endoscopic clip that will be detected by an antenna included in the custom-made sensing instrument in a similar fashion with the method exposed in the present work.

The success of this quest will move the field of laparoscopic surgical oncology toward an era of effective, personalized tumor detection, allowing precise identification of tumor location and its margins solely by the surgeon, during all steps of the surgical procedure and without compromising exposure of the operative field and thus coming one step closer to the final goal which is to offer a better perspective for the outcome of our patients.
